# Central Giant Cell Granuloma Misdiagnosed as Mandibular Osteomyelitis: A Case Report

**DOI:** 10.7759/cureus.107107

**Published:** 2026-04-15

**Authors:** Emmanouil Chatzipetros, Zafeiroula Yfanti, Konstantina-Eleni Alexiou, Christos Angelopoulos

**Affiliations:** 1 Department of Oral Diagnosis and Radiology, School of Dentistry, National and Kapodistrian University of Athens, Athens, GRC

**Keywords:** aggressive central giant cell granuloma, benign tumor, cone-beam computed tomography (cbct), osteolytic bone lesion, osteolytic tumor, osteomyelitis diagnosis, posterior mandible

## Abstract

This case report presents the diagnostic workflow of a patient initially suspected of osteomyelitis of the jaw who was ultimately diagnosed with central giant cell granuloma (CGCG). A 60-year-old male patient with no significant medical history presented with a painful intraoral swelling in the posterior region of the left mandible. The patient reported the extraction of the left mandibular first molar six months prior to presentation. Radiographic evaluation included periapical, panoramic, and cone-beam computed tomography (CBCT) imaging, which revealed an osteolytic lesion with ill-defined borders, as well as erosion and destruction of the cortical plates. Based on the clinical presentation and radiographic findings, the initial diagnosis was osteomyelitis of the mandible. Surgical removal of the lesion was performed, and subsequent histopathological examination unexpectedly established the diagnosis of CGCG. During the one-year follow-up period, the patient showed no evidence of recurrence. This case underscores the importance of a comprehensive, multidisciplinary diagnostic approach and highlights the need to maintain a broad differential diagnosis when evaluating destructive lesions of the jaws, as biopsy and histopathological examination remain essential for definitive diagnosis.

## Introduction

Osteomyelitis (OM) is an inflammation of the jawbones, involving both cortical and cancellous bone, usually due to odontogenic infections, trauma, post-surgical infections, hematogenous spread, and systemic conditions. It is classified as acute, chronic, or diffuse sclerosing OM [1]. OM presents with a combination of localized and systemic signs and symptoms, often following dental infections, trauma, or surgery. Patients typically experience deep, persistent, throbbing pain in the affected jaw, accompanied by facial swelling, erythema, and tenderness over the involved area, fever, malaise, lymphadenopathy, pus discharge, intraoral or extraoral sinus tract formation, tooth mobility, trismus, and in advanced cases, paresthesia or anesthesia of the affected region [1]. Radiographically, OM is characterized by the presence of osteolytic areas, sequestration, varying degrees of sclerosis, and periosteal new bone formation [2]. However, these clinical and radiographic features are not specific and may overlap with those of other inflammatory, reactive, or neoplastic lesions of the jaws, particularly in atypical presentations [1,2].

Central giant cell granuloma (CGCG) most commonly presents during the second and third decades of life, demonstrating a higher prevalence among women and exhibiting a predilection for the mandible over the maxilla. Although the etiology of CGCG is not fully understood, it is generally regarded as a reactive lesion, possibly related to local trauma, infection, or hormonal factors [3]. CGCG demonstrates a wide range of radiographic features, varying from well-defined, expansile lesions to ill-defined, aggressive lesions, with or without accompanying cortical expansion [3-5]. It may also include specific radiographic features such as multilocular or unilocular appearance, “soap bubble” or “honeycomb” patterns, root resorption, and tooth displacement [4,5]. In its more aggressive or atypical forms, CGCG may present with features such as pain, rapid growth, and cortical bone destruction, which can closely resemble those observed in inflammatory conditions such as OM [2].

OM can be difficult to distinguish from other inflammatory conditions, as well as less common entities such as cysts and neoplasms, posing a diagnostic challenge, particularly in atypical cases. While advanced imaging modalities like cone beam computed tomography (CBCT) enhance diagnostic precision, histopathological analysis remains essential for definitive diagnosis [6]. Importantly, the presence of an apparent odontogenic cause, such as recent tooth extraction or infection, may further bias the clinician toward a diagnosis of OM, potentially overlooking less common entities such as CGCG [1,3].

This case report presents the diagnostic workflow of a patient initially suspected of OM of the jaw, who was ultimately diagnosed with CGCG. It aims to highlight the diagnostic overlap between OM and CGCG, a potential but often overlooked source of misdiagnosis, particularly in older patients.

## Case presentation

A 60-year-old male patient with no significant medical history presented with a painful, suppurative swelling localized to the posterior region of the left mandible. The patient reported that the swelling had been present for several weeks and had progressively worsened. His dental history included the extraction of the left mandibular first molar approximately six months earlier, performed due to a symptomatic condition, although details of the initial pathology were not available. Shortly after extraction, the patient developed swelling, which led to the initial consultation.

**Figure 1:**
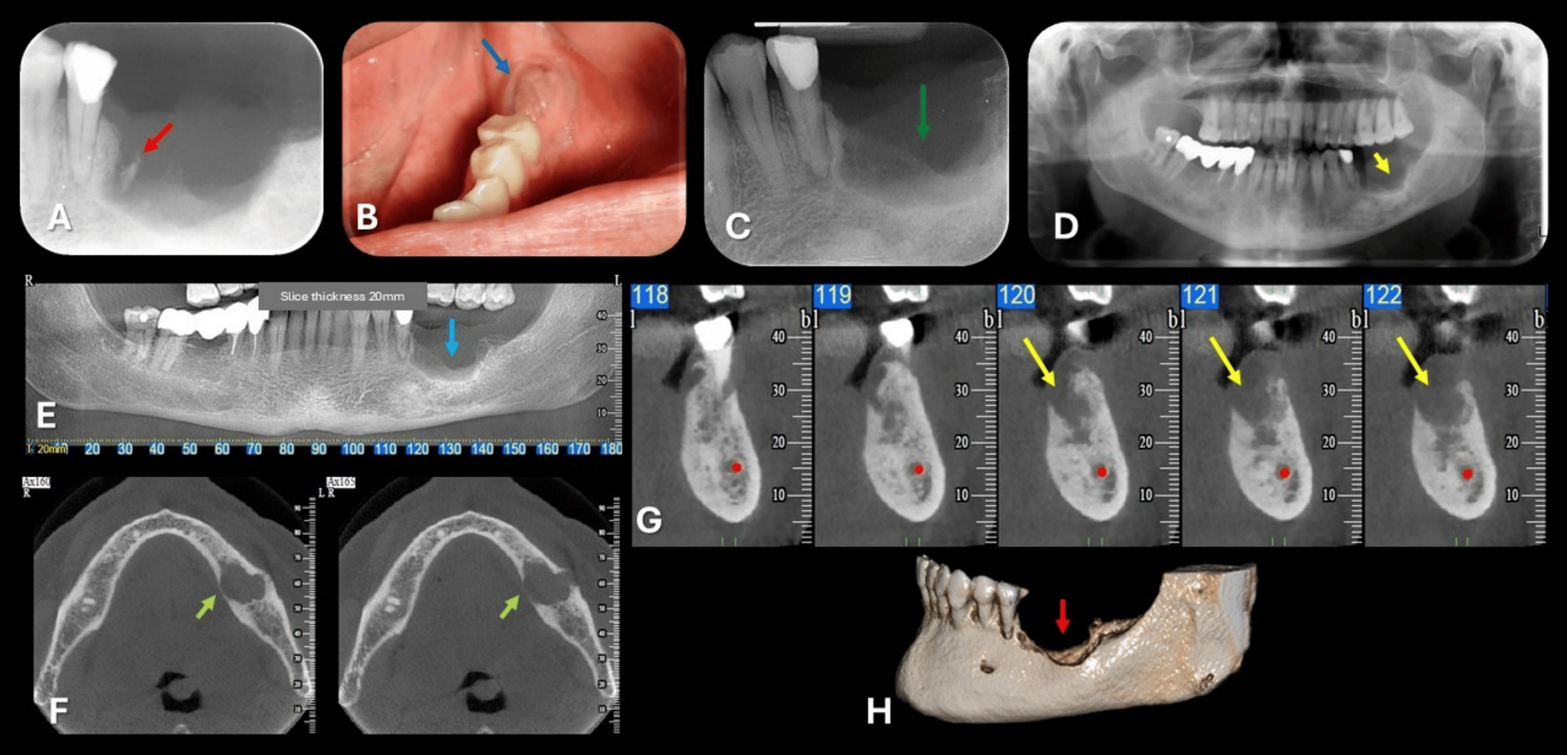
Clinical and radiographic findings of the lesion. (A) Periapical radiograph showing a retained root fragment (red arrow) at the extraction site, suggestive of local irritation and possible infection. (B) Clinical image showing a well-defined, red, granular mass with elastic consistency and a pedunculated base located on the gingival tissue adjacent to the extraction site (blue arrow). (C) Periapical radiograph of the left mandibular first molar region (green arrow). (D) Panoramic radiograph demonstrating a radiolucent lesion in the left mandibular first molar region (yellow arrow). (E) Cone-beam computed tomography (CBCT) panoramic reconstruction showing an osteolytic lesion with ill-defined borders and cortical plate erosion (blue arrow). (F) Axial CBCT images of the lesion (green arrows). (G) Cross-sectional CBCT images (yellow arrows). (H) Three-dimensional CBCT reconstruction highlighting the lesion (red arrow).

A periapical radiograph revealed a retained root fragment at the extraction site, presumably causing local irritation and infection (Figure 1A). According to the patient’s history, surgical removal of the retained root fragment was performed along with antibiotic regimen, resulting in temporary symptom relief. However, the swelling subsequently recurred, prompting further evaluation.

Upon clinical examination, a well-defined, red, granular mass with elastic consistency and a pedunculated base was identified on the gingival tissue adjacent to the extraction site (Figure 1B). Purulent discharge was evident, consistent with active inflammation. No associated systemic symptoms were reported. Radiographic examinations followed, including periapical (Figure 1C), panoramic (Figure 1D), and CBCT imaging (Figures 1E-1H). CBCT revealed an osteolytic lesion with ill-defined borders, erosion, and destruction of cortical plates (Figures 1E-1H). 

Given the absence of systemic signs and symptoms suggestive of active infection, such as fever, malaise, or lymphadenopathy, no hematological tests, infection markers, or microbiological investigations were initially deemed necessary. Overall, the clinical and radiographic features oriented the initial differential diagnosis toward an inflammatory/reactive etiology. The acute clinical presentation, characterized by pain, suppuration, and its association with a recent extraction and retained root fragment, favored an inflammatory process over a primary benign intraosseous lesion such as CGCG, which typically affects younger individuals, in contrast to the 60-year-old patient in the present case. Similarly, although the radiographic appearance of an ill-defined osteolytic lesion with cortical destruction could raise suspicion for malignancy, this possibility was considered less likely at the initial stage due to the absence of characteristic features, such as paresthesia, regional lymphadenopathy, or systemic manifestations, along with the presence of a localized infection and purulent discharge, which supported an inflammatory etiology. Furthermore, the temporary symptom relief observed following antibiotic therapy was not consistent with the expected behavior of a malignant process.

**Figure 2:**
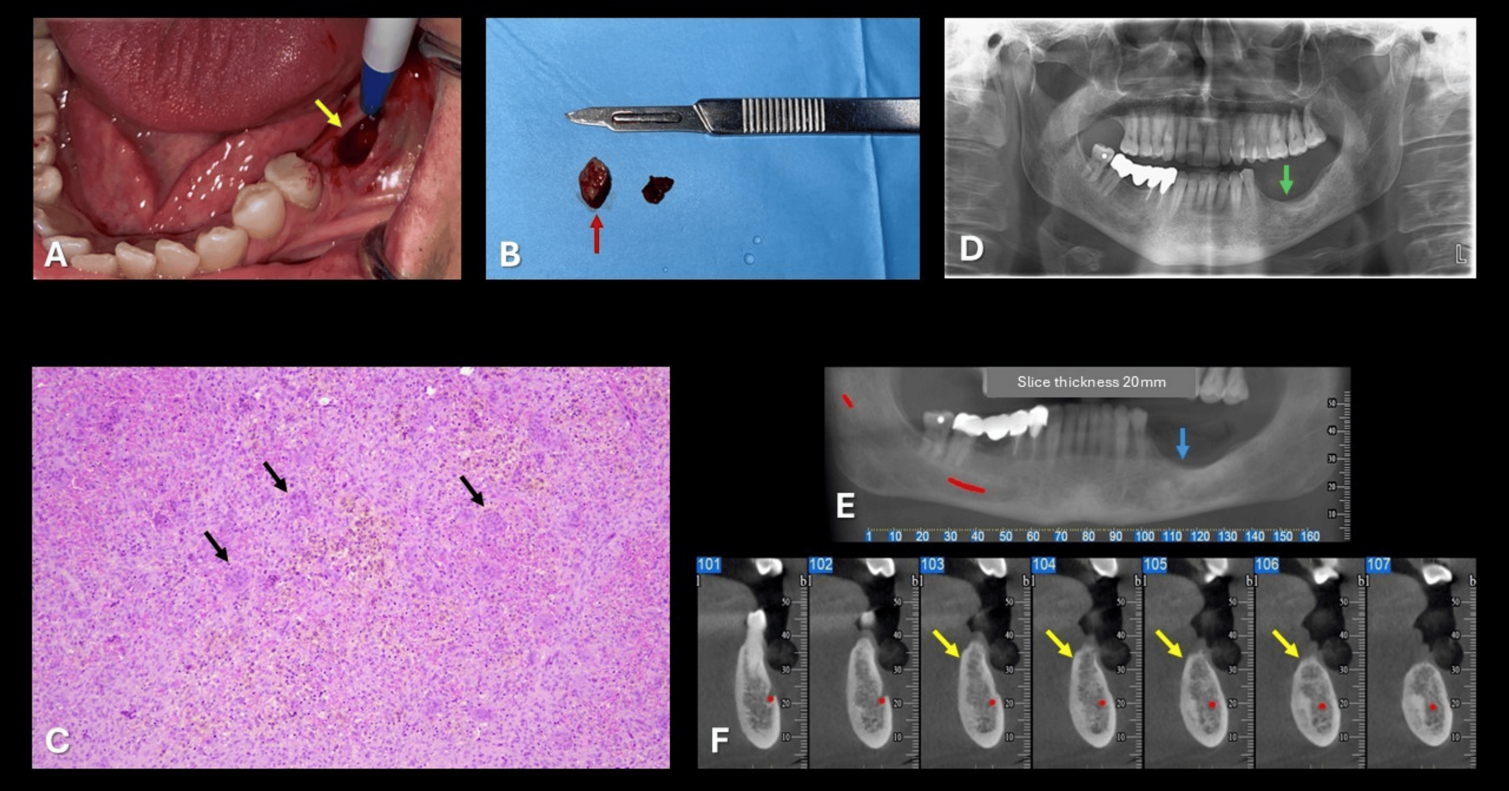
Intraoperative, histopathological, and radiographic follow-up findings. (A) Intraoperative view of the lesion (yellow arrow). (B) Excised tissue specimen (red arrow) submitted for histopathological examination. (C) Histopathological findings showing clusters of osteoclast-type multinucleated giant cells (black arrows) within a vascularized fibrous stroma, with areas of hemorrhage and hemosiderin deposition (hematoxylin and eosin stain, original magnification × 20). (D) One-year follow-up panoramic radiograph demonstrating bone healing in the left mandibular first molar region (green arrow). (E) Cone-beam computed tomography (CBCT) panoramic reconstruction showing healing of the previously identified osteolytic lesion at the margin of the edentulous alveolar ridge (blue arrow). (F) CBCT cross-sectional images highlighting bone healing (yellow arrows).

Based on the clinical and radiographic findings, the initial diagnosis was OM of the mandible. Nevertheless, a biopsy with subsequent histopathological evaluation was deemed essential to establish and confirm the initial diagnosis. Surgical excision of the lesion (Figures 2A, 2B) was performed, and histopathological examination (Figure 2C) unexpectedly revealed a diagnosis of CGCG. During the one-year follow-up, the patient demonstrated no evidence of disease recurrence (Figures 2D-2F).

**Figure 3:**
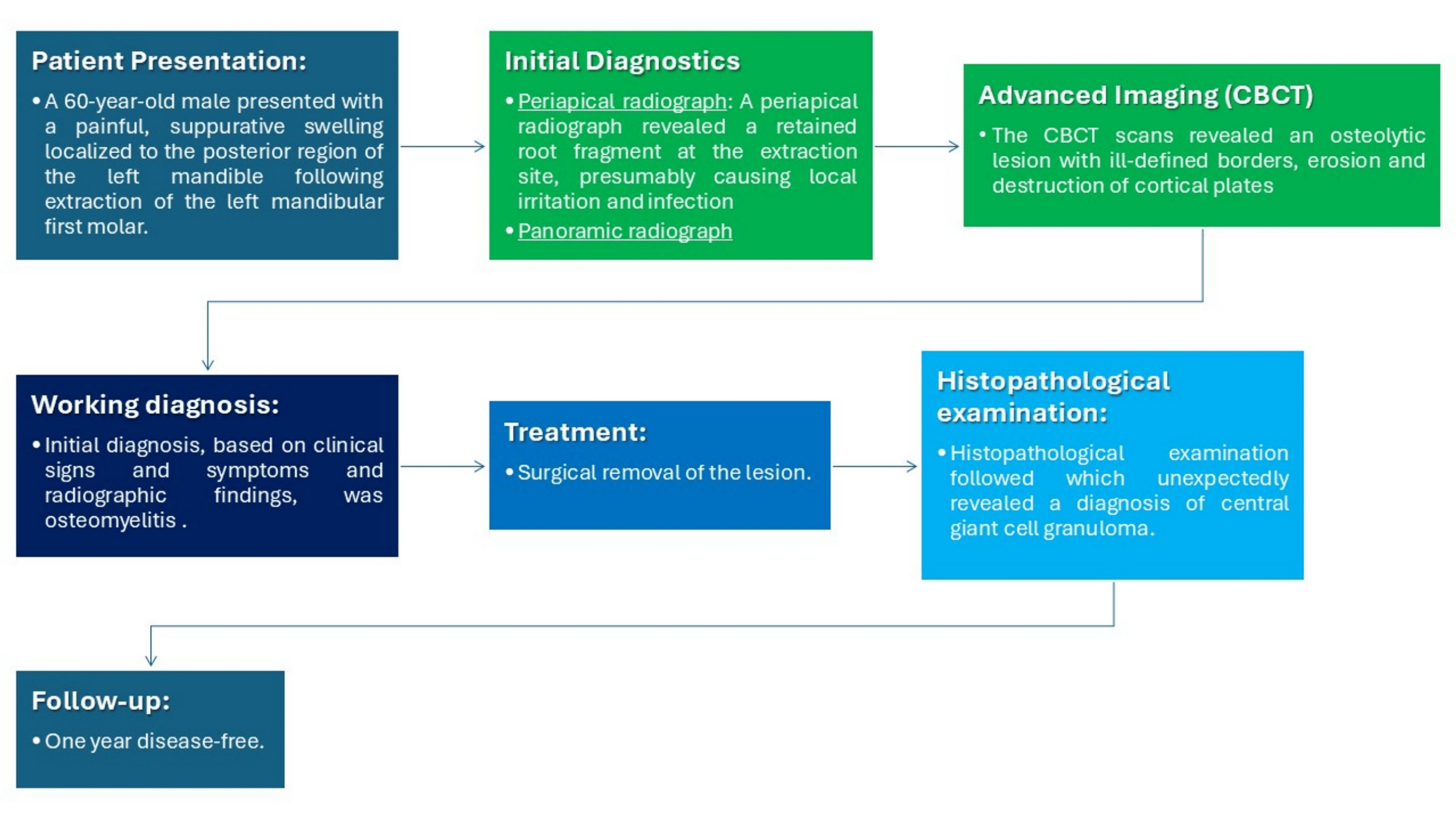
Timeline of clinical presentation, imaging findings, and treatment. Figure created by authors using Microsoft PowerPoint (Microsoft Corporation, Redmond, Washington, United States)

The entire clinical course is depicted in Figure 3. 

## Discussion

This case involved a 60-year-old male patient who was initially misdiagnosed with OM in the posterior mandible due to atypical clinical and radiographic features. Surgical excision and histopathological analysis later confirmed the correct diagnosis: CGCG.

CGCG is recognized as a locally aggressive lesion of unknown cause. It was first described as a reparative process of bone occurring in response to trauma, inflammation, or hemorrhage within the bone marrow [3]. Although historically described as a reparative process, the pathogenesis of CGCG remains controversial, with ongoing debate regarding its reactive versus neoplastic nature [7]. CGCG demonstrates a broad spectrum of clinical and biological behavior, ranging from indolent, asymptomatic lesions with low recurrence potential (non-aggressive form) to aggressive, rapidly enlarging lesions characterized by pain, local bone destruction, root resorption, tooth displacement, and a high risk of recurrence (aggressive form) [3,7].

The lesion can occur at any age, although it is more common in individuals under 30 years of age and shows a female predominance [8]. Mandibular involvement is more common than maxillary (2.67:1) [8], with the anterior mandible being the most frequently affected site [3]. Multiple lesions are rare but often linked to systemic conditions like neurofibromatosis type 1 and Noonan syndrome [8]. Unlike previous studies, the current case involved a 60-year-old male patient with an unremarkable medical history, presenting with a painful, suppurative swelling in the posterior left mandible. Furthermore, a broad spectrum of radiographic features has been reported for the mandibular CGCG: well-defined borders with a scalloped margin, moderate cortication, hypodense appearance of the internal structures, unilocular lesion with no septa, both displacement and resorption on adjacent teeth [3]. In this case, the CBCT scans revealed an osteolytic lesion with ill-defined borders, erosion, and destruction of cortical plates. These features, particularly in aggressive forms, may overlap with inflammatory conditions, as cortical destruction and ill-defined borders are not exclusive to neoplastic processes, thereby contributing to potential diagnostic ambiguity.

On the other hand, jaw OM often manifests as localized pain, swelling, erythema, purulent discharge, fistula or sinus tract formation, sequestrum and involucrum development, tooth mobility, and, in some cases, numbness or paresthesia due to nerve involvement [1,9,10]. The jawbones’ complex anatomy and relatively limited blood supply, especially in the posterior mandible, contribute to a greater susceptibility to infection, impaired healing, and increased risk of chronicity due to reduced antibiotic penetration [9]. OM is radiographically characterized by the presence of osteolytic lesions, sequestra, areas of sclerosis, and periosteal new bone formation [2]. Cancellous bone destruction and osteolysis were the predominant radiographic findings observed [2]. The most common site of OM in the jaws is the posterior region/body of the mandible [11]. In this case, the patient's dental history revealed the extraction of the left mandibular first molar, accompanied by localized swelling and the presence of a retained root fragment at the extraction site. These findings may represent a potential etiological factor in the pathogenesis of OM. Importantly, the radiographic presentation in this case, characterized by ill-defined osteolysis and cortical disruption, strongly supported an initial diagnosis of OM, especially when interpreted in conjunction with the patient’s history of recent tooth extraction, highlighting how clinical context may influence radiographic interpretation. However, these imaging features are not pathognomonic, and their overlap with aggressive CGCG underscores the limitations of relying solely on radiographic criteria for definitive diagnosis.

On radiological examination, CGCG must be considered in the differential diagnosis of other lesions featuring giant cells, such as giant cell tumor, cherubism, brown tumor associated with hyperparathyroidism, and aneurysmal bone cyst [8]. However, in clinical practice, the differential diagnosis may be further complicated when CGCG presents with atypical or aggressive imaging features. OM has not been widely reported as part of the differential diagnosis of CGCG in existing literature [2]. This highlights a potential gap in diagnostic consideration, as overlapping clinical and imaging features, such as pain, swelling, and osteolytic bone destruction, may lead to diagnostic misinterpretation, particularly in atypical presentations. In this context, the present case emphasizes that radiographic findings, although highly suggestive, should be interpreted with caution, and definitive diagnosis requires correlation with histopathological evaluation. The discrepancy between the initial radiographic impression and the final diagnosis further illustrates the importance of a systematic and multidisciplinary diagnostic approach. Nevertheless, limitations exist: although CBCT was essential for initial assessment, definitive diagnosis ultimately requires histopathological confirmation. Furthermore, the findings from a single rare and atypical case may not be broadly generalizable.

On histopathological examination, CGCG should be differentiated from other osteolytic jaw lesions containing giant cells, including brown tumor of hyperparathyroidism, giant cell tumor, and non-ossifying fibroma, as well as entities such as cherubism and aneurysmal bone cyst, which may present with similar histopathological features [7,12]. A limitation of the present case is the absence of biochemical investigations, which are important for excluding a brown tumor of hyperparathyroidism. This limitation is acknowledged, as the histopathological overlap between CGCG and brown tumor may preclude definitive distinction based on morphology alone. However, in the present case, the lesion presented as a solitary intraosseous mandibular lesion, without clinical or medical history suggestive of an underlying systemic metabolic bone disease. Taking into account the localized nature of the lesion, the absence of multifocal skeletal involvement, and the overall clinicopathological correlation, the findings were considered most consistent with CGCG. Based on these considerations, surgical excision was performed as the treatment of choice. At the one-year follow-up, no evidence of recurrence was observed, further supporting the diagnosis of a localized CGCG with favorable clinical behavior.

## Conclusions

This case underscores the significance of a comprehensive, multidisciplinary diagnostic approach and highlights the necessity of maintaining a broad differential diagnosis when assessing destructive lesions of the jaws; biopsy and histopathological analysis are necessary for definitive diagnosis. Importantly, this case demonstrates that radiographic findings suggestive of OM may mask underlying non-infectious lesions such as CGCG, particularly in the presence of an apparent odontogenic trigger. Clinicians should therefore exercise caution when interpreting imaging findings in isolation and consider early histopathological evaluation in atypical or non-resolving cases. These observations provide a practical diagnostic insight that may assist clinicians in avoiding similar misinterpretations in future cases.
